# Oral *P. gingivalis *infection alters the vascular reactivity in healthy and spontaneously atherosclerotic mice

**DOI:** 10.1186/1476-511X-10-80

**Published:** 2011-05-17

**Authors:** Raquel B Pereira, Elisardo C Vasquez, Ivanita Stefanon, Silvana S Meyrelles

**Affiliations:** 1 Department of Physiological Sciences, Health Sciences Center, Federal University of Espirito Santo, Vitoria, ES, Brazil

## Abstract

**Background:**

Considering that recent studies have demonstrated endothelial dysfunction in subjects with periodontitis and that there is no information about vascular function in coexistence of periodontitis and atherosclerosis, we assessed the impact of oral inoculation with the periodontal pathogen *Porphyromonas gingivalis *on vascular reactivity in healthy and hypercholesterolemic apolipoprotein E-deficient (ApoE) mice. *In vitro *preparations of mesenteric arteriolar bed were used to determine the vascular responses to acetylcholine, sodium nitroprusside and phenylephrine (PE).

**Results:**

Alveolar bone resorption, an evidence of periodontitis, was assessed and confirmed in all infected mice. Acetylcholine- and sodium nitroprusside-induced vasorelaxations were similar among all groups. Non-infected ApoE mice were hyperreactive to PE when compared to non-infected healthy mice. *P gingivalis *infection significantly enhanced the vasoconstriction to PE in both healthy and spontaneous atherosclerotic mice, when compared to their respective controls.

**Conclusions:**

This study demonstrates that oral *P gingivalis *affects the alpha-adrenoceptor-mediated vascular responsiveness in both healthy and spontaneous atherosclerotic mice, reinforcing the association between periodontitis and cardiovascular diseases.

## Background

Periodontal disease is a chronic inflammatory disease that affects the gum tissue and other structures supporting the teeth. It begins as gingivitis and, if left untreated, can progress to periodontitis, where destruction of connective tissue attachment and alveolar bone can lead to tooth loss. Beyond its local effects, periodontal disease may also interfere with other systems of the body, and many epidemiological studies have associated periodontitis with atherosclerosis. However, a causal relation between both diseases remains controversial [1].

Investigators have proposed that periodic transient bacteremia, which leads to invasion of vascular cells and increase of the levels of circulating cytokines, accelerates the atherogenic process [2]. Also, it has been reported that *P gingivalis*, the periodontopathogen associated with the most common form of periodontal disease, accelerates atheroma formation [3-5], increases systemic inflammatory markers [4,6-8], invades endothelium and vascular smooth muscle cells [9,10] and appears to alter endothelial function [11-15].

Endothelial dysfunction appears to be an early event in the development of atherosclerosis [16] and also predicts plaque instability [17]. Support for the clinical importance of vascular reactivity and endothelial dysfunction is provided by several studies demonstrating increased cardiovascular disease risk in patients with vascular dysfunction in coronary and peripheral arteries [17-22].

On the basis of these observations, we aim to investigate whether oral *P gingivalis *infection alters the vascular responsiveness in spontaneous atherosclerosis in apolipoprotein E-deficient (ApoE) mice compared with C57BL/6 (C57) wild-type mice.

## Methods

### Experimental groups

Experiments were conducted on adult (30-week-old) male C57 and ApoE mice from the Laboratory of Transgenes and Cardiovascular Control of the Federal University of Espirito Santo. Animal care and treatment were approved by the institutional Ethics Committee for Use of Animals (CEUA-Emescam, Protocol # 020/2007) and were conducted in conformity with institutional guidelines, in compliance with international laws and policies. C57 mice were used as animals systemically healthy. C57 and ApoE animals were randomly divided in control (Ct) or infected (Pg) groups: C57 Ct, C57 Pg, ApoE Ct, ApoE Pg.

### Preparation of bacterial culture and oral infection

*P gingivalis *strain ATCC 33277 was obtained from the Collection of Microorganisms of Reference (INCQS, Fiocruz, Brazil) and cultured in blood-agar supplemented with hemin/menadione, under an anaerobic condition [23]. At age of 18 weeks randomly selected mice were infected with *P gingivalis *as follows. 10^9 ^CFU of live bacteria (optical density of ≈ 0.8 at 660 nm) in 100 μL of PBS with 2% carboxymethylcellulose was administered via oral topical application three times at 2-day intervals. Control mice received carboxymethylcellulose in PBS. Infected mice were kept in microisolated cages (Beiramar, Brazil).

### Cholesterol and systemic inflammation analysis

At 30 weeks of age, under anesthesia with thiopental (40 mg/kg i.p.), mice were subjected to the axillary plexus isolation and samples of blood were obtained by punction [24]. For each mouse two blood samples were collected. Serum was obtained to determine the levels of plasma total cholesterol by chromogenic assays (Bioclin, Brazil). Blood was used to perform leucogram (Beckman Coulter MAXM HMX, USA). Systemic host inflammatory response or systemic inflammation was assessed by neutrophils/lymphocytes ratio [25].

### *"In Vitro" *preparation of the mesenteric arteriolar bed

Isolated mesenteric arteriolar beds were obtained 12 weeks after *P gingivalis *inoculation of the animals and after blood samples collection. Briefly, the superior mesenteric artery was cannulated (PE 50, Becton Dickinson, Sparks, MD, USA), and the intestine was immediately severed from the body. The mesenteric vascular bed was perfused at a constant flow of 2 mL/min with oxygenated (mixture of 95% O_2 _and 5% CO_2_, 37°C) physiological salt solution (130 NaCl, 4.7 KCl, 1.6 CaCl_2_◦2H_2_O, 1.8 KH_2_PO_4_, 4.7 MgSO, 1.17 H_2_O, 14.9 NaHCO_3_, 0.026 EDTA, and 11.1 glucose; mmol/L), using a roller pump (Harvard Apparatus, USA). The bowel was removed to prevent the return of the Krebs' solution through the venous system. After passing through the vascular bed, the perfusate was artificially drained out from the preparation. After a 40-min stabilization period, the experimental protocol was initiated. Mean perfusion pressure (MPP) was measured; as a result of maintenance of a constant flow, changes in the MPP represented changes in vascular resistance. The vasoconstrictor response was assessed by stimulation of α-adrenoceptors evoked by phenylephrine (PE; 0.001-300 μg; Sigma-Aldrich). To study endothelium-dependent and endothelium-independent vasodilation, responses to acetylcholine (ACh; 10^-10 ^to 10^-3 ^mol/L; Sigma-Aldrich) and sodium nitroprusside (SNP; 10^-10 ^to 10^-3 ^mol/L; Sigma-Aldrich) respectively were determined as percentages of reductions in the precontractions induced by 10^-5 ^mol/L PE (concentration that induces 60% to 80% of the maximal effect).

### Quantification of atherosclerotic lesion area

After bowel removal the heart was perfused through right atrium with 50 mL of PBS (0.1 M; pH 7.4), following by 50 mL of formaldehyde (4%). Aorta was removed and stored in buffered fixative solution, and cryostat sections were prepared, as previously described [26]. Atherosclerotic lesion area was quantified using a microscope (Olympus, Japan) interfaced with a videocamera (Hitachi, Japan) and an image analysis system (Image J, USA). Mean lesion area per mouse was calculated by an investigator blinded to the experimental protocol and was expressed as μm^2^.

### Quantification of alveolar bone loss

Alveolar bone between first and second molars of the left mandible was assessed by a morphometric method. Briefly, after 15 minutes in boiling water mandible was mechanically defleshed, exposed overnight in 3% hydrogen peroxide and immersed in bleach for 1 minute. The bone level, that is, the distance from the cementum-enamel junction (CEJ) to the alveolar bone crest (ABC) was measured under a dissecting microscope (× 40) with a total of 2 measurements per mouse. The measurements were repeated two times per site in random and blinded protocols. Because a greater distance from the CEJ-ABC indicates less alveolar bone, bone levels were converted to relative amounts of bone by the following calculation: 1/distance from CEJ-ABC [23]. The values were normalized by converting bone values of each mouse to a percentage of the mean value for respective control mice group.

### Statistical Analysis

Vascular responses are given as percentage of dilation relative to the PE-induced preconstriction level. Values are expressed as means ± SEM. For each dose-response curve, the maximum effect (*E*_max_) and the dose of agonist that produced one-half of *E*_max _(ED_50_) were estimated using nonlinear regression analysis (GraphPad Software Inc., San Diego, CA). The sensitivity of the agonists is expressed as pED_50_. One- and two-way ANOVA, followed by Bonferroni *t *test, or unpaired Student's *t *test when appropriated, were used for statistical analyses. *p *< 0.05 was considered statistically significant.

## Results

### Alveolar bone loss produced by oral infection with *P gingivalis*

**Figure 1:**
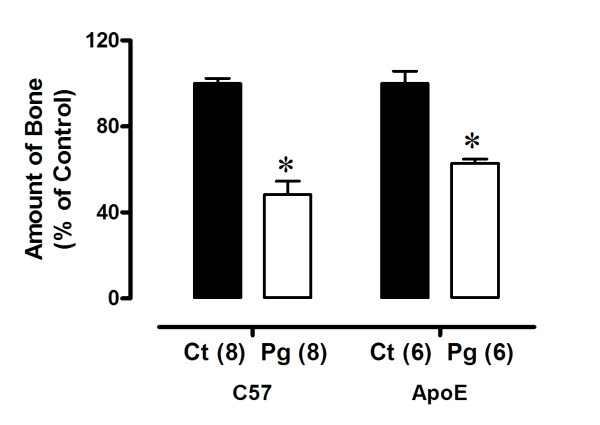
**Alveolar bone loss produced by oral infection with *P gingivalis *in C57 and ApoE mice**. C57 represents animals systemically healthy and ApoE represents animals with spontaneous atherosclerosis. The relative amounts of bone are significantly less in infected (Pg) than in control (Ct) mice. Values are means ± SEM. * *p *< 0.05 vs Ct group.

To determine the degree of local periodontal destruction, we dissected the mandibles after euthanasia and measured alveolar bone loss. As summarized in Figure 1, infected mice displayed significantly increased alveolar bone loss compared with the respective control, indicated by a decrease in relative amounts of bone in infected mice (C57 Ct; 100 ± 2 versus C57 Pg; 48 ± 6%; ApoE Ct: 103 ± 6 versus ApoE Pg: 63 ± 2%, respectively; *p *< 0.05 for all comparisons).

### Effects of the oral *P gingivalis *infection on serum cholesterol levels and systemic inflammatory response

**Figure 2:**
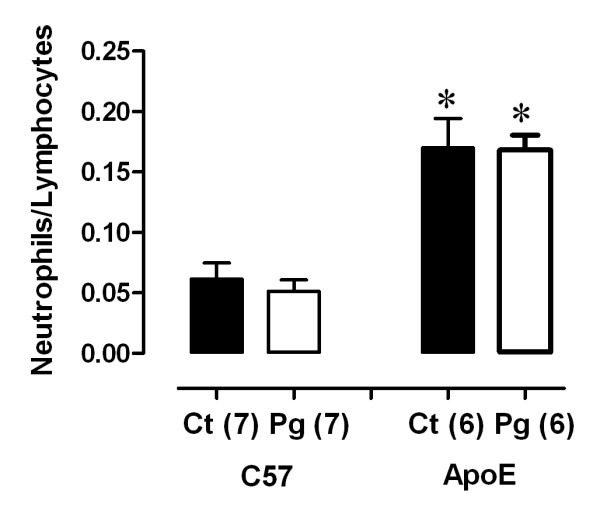
**Oral *P gingivalis *infection and systemic inflammation**. Atherosclerotic mice have increased neutrophils/lymphocytes ratio when compared to C57. Ct: control animals; Pg: infected animals. *p < 0.05 vs. C57.

We investigated if the oral infection with *P gingivalis *modulated established risk factors for atherogenesis in this murine model. No differences in total plasma cholesterol were observed between infected and control groups in both C57 (Ct: 78 ± 6 versus Pg: 85 ± 3 mg/dL) and ApoE groups (Ct: 523 ± 64 versus Pg: 629 ± 41 mg/dL) groups (Table 1). When systemic inflammation was analyzed we observed an increase (~ 3-fold) in neutrophils/lymphocytes ratio from mice with atherosclerosis compared to C57 in both control (ApoE: 0.17 ± 0.01 versus C57: 0.06 ± 0.01; *p *< 0.05) and infected (ApoE: 0.17 ± 0.02 versus C57: 0.05 ± 0.001; *p *< 0.05) animals (Figure 2). However, oral *P gingivalis *inoculation did not change (*p *> 0.05) the systemic inflammatory status in both healthy and atherosclerotic mice, when compared to their controls (Figure 2). Body weight was also monitored and was similar among all groups (Table 1).

**Table 1:** Effects of Oral Infection with P gingivalis on Serum Cholesterol and Body Weight

Parameters	C57 Ct (8)	C57 Pg (8)	ApoE Ct (7)	ApoE Pg (6)
Body weight (g)	35 ± 0.6	33 ± 0.6	34 ± 0.8	33 ± 1.5
Serum cholesterol (mg/dL)	78 ± 6	85 ± 3	523 ± 64^†^	629 ± 41^§^

Values are mean ± SEM.

^† ^vs C57 CT; ^§ ^vs C57 Pg; *p *< 0.05.

### Effects of the oral *P gingivalis *infection on atherosclerotic lesion area

**Figure 3:**
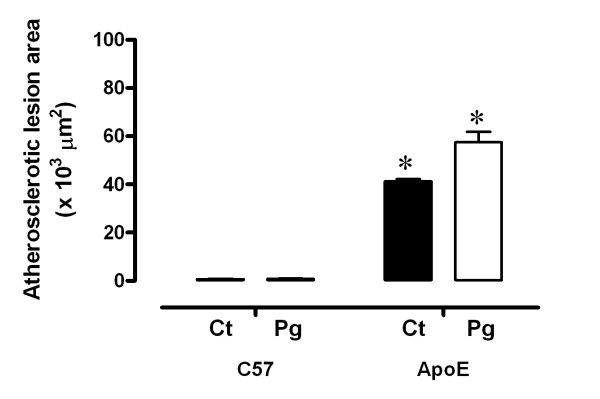
**Oral *P gingivalis *infection and atherosclerotic lesion areas**. Atherosclerotic mice have increased lesion areas when compared to C57. Oral infection does not alter the mean atherosclerotic lesion areas. Ct: control animals; Pg: infected animals. *p < 0.05 vs. C57.

To evaluate if the oral infection with *P gingivalis *amends the progression of atherosclerotic lesions, morphometric analyses were used and demonstrated that there were no differences (*p *> 0.05) in mean atherosclerotic lesion area in infected mice compared with controls in both C57 (Pg: 0.75 ± 0.2 versus Ct: 0.66 ± 0.2 μm^2 ^× 10^3^) and ApoE (Pg: 57.5 ± 4.3 versus Ct: 41.25 ± 0.9 μm^2 ^× 10^3^) groups (Figure 3).

### Effects of the oral *P gingivalis *infection on mesenteric arteriolar bed responsiveness

**Figure 4:**
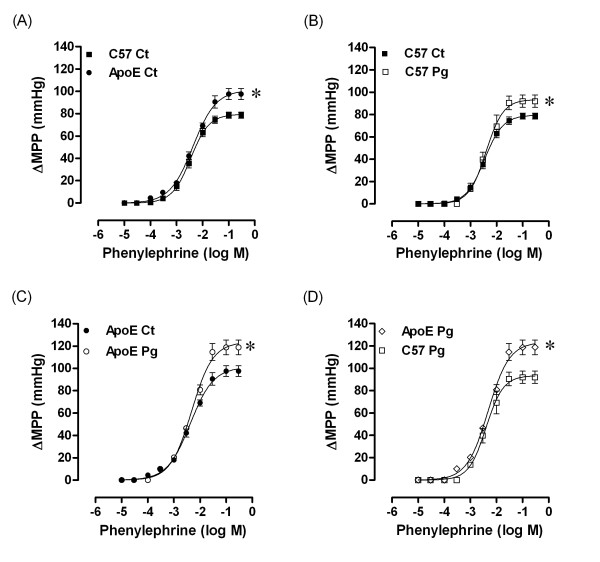
**Phenylephrine dose-response curves in mesenteric arteries of C57 and ApoE mice, 12 weeks after *P gingivalis *oral inoculation**. A: Effect of atherosclerosis on vasoconstriction; B: Effects of oral infection on vascular contractile response in systemically healthy mice; C: Effects of oral infection on vascular contractile response in mice with atherosclerosis; D: Difference on vascular contractile response between animals systemically healthy and with atherosclerosis. Ct: control animals; Pg: infected animals. *p < 0.05.

We examined the effects of *P gingivalis *oral infection on mesenteric arteriolar bed responsiveness to phenylephrine, acetylcholine and sodium nitroprusside. As summarized in Figure 4 (panel A) the spontaneous atherosclerotic mice were hyperreactive to phenylephrine when compared to systemically healthy mice (ApoE Ct: 98 ± 5 mmHg versus C57 Ct: 79 ± 3 mmHg; *p *< 0.05). Periodontitis caused by *P gingivalis *oral inoculation enhanced (*p *< 0.05) the maximal response to phenylephrine in all groups when compared to the respective control group in both C57 (Pg; 92 ± 6 versus Ct: 79 ± 3 mmHg) and ApoE (Pg: 119 ± 7 versus Ct: 98 ± 5 mmHg) groups (Figure 4B and 4D). Figure 4 (panel D) shows the preservation of the vascular hyperreactivity to phenylephrine of atherosclerotic mice (119 ± 7 mmHg) compared to healthy mice (92 ± 6 mmHg; *p *< 0.05) even after *P gingivalis *infection. Despite of changes in vasoconstrictor responses, neither endothelium-dependent nor endothelium-independent vasodilations were changed by *P gingivalis *oral infection (values shown in Table 2).

**Table 2:** Effects of Oral Infection with P gingivalis on Vascular Reactivity to PE, ACh and SNP

Parameters	C57 Ct (8)	C57 Pg (8)	ApoE Ct (7)	ApoE Pg (6)
PE				
E_max _(mmHg)	79 ± 2.7	92 ± 5.5*	98 ± 5^†^	119 ± 7 *^‡^
pED_50_	2.5 ± 0.09	2.37 ± 0.06	2.40 ± 0.07	2.3 ± 0.05
ACh				
E_max _(% of relaxation)	76 ± 2.3	74.5 ± 1.4	75.5 ± 2.9	75 ± 3
pED_50_	6.15 ± 0.17	6.25 ± 0.1	6.12 ± 0.27	6 ± 0.18
SNP				
E_max _(% of relaxation)	92 ± 1.3	89 ± 0.8	91 ± 2.5	92 ± 2
pED_50_	6.6 ± 0.07	6.71 ± 0.04	6.90 ± 0.10	6.7 ± 0.08

Values are mean ± SEM.

*Pg versus Ct; ^†^versus C57 Ct, ^‡ ^versus C57 Pg; *p *< 0.05.

## Discussion

The present study was designed to test whether oral challenge with an established periodontal pathogen amends vascular responsiveness in a murine model of spontaneous atherosclerosis. Surprisingly, our data show that oral *P gingivalis *infection not only increases the vascular contractile response to phenylephrine in atherosclerotic mice but also in systemically healthy mice. The ApoE model of atherosclerosis used in this study is well established [27,28]. In agreement with previous studies we detected high levels of plasma cholesterol in ApoE mice fed a standard chow diet [28-31]. We also noted that the systemic inflammation observed in this study is consistent with the fact that atherosclerosis is a systemic inflammatory disease [32]. An important finding of this study is that oral *P gingivalis *inoculation did not change the systemic inflammatory status in atherosclerosis, a result consistent with that described by Miyamoto et al. [33]. In healthy animals it was not detected systemic inflammatory response induced by oral *P gingivalis *infection, although some authors have reported an increase in systemic inflammatory markers such as C-reactive protein, interleukin 6 and neutrophils in subjects with periodontitis [6-8]. Therefore, more studies are necessary to elucidate this controversial data. One consideration and limitation is that in the present study we investigated the effects of only one periodontal pathogen, while human periodontitis encloses several microorganisms.

The oral *P gingivalis *infection did not influence the serum cholesterol levels. This result is in agreement with those found in mice infected with *P gingivalis *[4] and humans [11] with periodontitis. Although literature reports an increase in the area of atherosclerotic lesion caused by *P gingivalis *in animal models [4,5,33,34], in this study we did not find changes in the atherosclerotic lesion area. We attribute this difference to different methodologies used in the studies. Li et al. [5] reported increase in the area of injury only after 14 intravenously weekly inoculations of *P gingivalis *(10^7 ^CFU) in atherosclerotic mice. Animals inoculated for 10 weeks did not show any change in the area of plaque [5]. In our study we performed only three inoculations, and to mimic normal conditions for which periodontal pathogens could reach the circulatory system, the inoculation of *P gingivalis *was oral topical, and not intravenously as described by those authors. Lalla et al. [4] also observed increased aortic lesions in ApoE mice after 15 inoculations (10^12 ^CFU) with *P gingivalis*. Again, the number of inoculations in that study was high and was performed by oral gavage and anal topical application; the latter mode used to establish a cycle of oral reinfection, because mice use to be coprophagic. In the present study, however, the extent of the injury may not have changed because we used smaller burden of pathogens (10^9 ^CFU), the inoculations were less sparse and smaller in number, and because we used only oral topical inoculation, that is more compatible with periodontitis in humans. Despite of a great difficulty in establishing a model of periodontitis similar to periodontal disease in humans, *P gingivalis *inoculation reproduces the periodontal tissue destruction found in humans, making this model well accepted to study periodontitis.

Endothelial dysfunction has been considered one of the early steps in atherosclerosis [32]. Although endothelial dysfunction has been frequently considered when an impaired endothelium-dependent vasodilation is observed, the localized modulation of vascular endothelium to a nonadaptive functional state can be termed as endothelial dysfunction [35]. In ApoE mice, endothelial dysfunction, taken as an impaired endothelium-dependent dilation, is controversial. Endothelial dysfunction can be detected or not in ApoE mice depending on the type of diet, age, gender, and type of vessel [36-45]. Recently it was shown in the mesenteric vascular bed from male ApoE mice vascular dysfunction, characterized by increased pressor responsiveness to norepinephrine, despite of normal endothelium-dependent and -independent relaxations [44]. Similarly, we observed a hyperreactive response to phenylephrine in atherosclerotic animals without changes in endothelium-dependent and -independent vasodilations to acetylcholine and sodium nitroprusside, respectively. At present, the mechanism by which hypercholesterolemia alters vascular responsiveness in mesenteric arteriolar bed is unknown and further studies will try to elucidate this issue.

Interestingly, the hyperreactivity to the α-adrenoceptor agonist in ApoE mice infected with *P gingivalis *was exacerbated when compared with noninfected ApoE animals. In the systemically healthy mice, the response to phenylephrine was also increased, but if we compare its maximal responses, the hyperreactivity was more pronounced in ApoE mice. The mechanism by which oral *P gingivalis *infection interferes with the reactivity to phenylephrine is unknown. However, based on the finding of an increased production of endothelin in crevicular fluid in subjects with periodontitis [46] and that the actions of endothelin include cell proliferation, migration and contraction [47], we speculate that one of the possible mechanisms by which periodontitis leads to exacerbated pressor response to α-adrenoceptor agonists could be the increase of systemic levels of endothelin.

## Conclusions

In conclusion, in the present study we demonstrate that oral *P gingivalis *infection amplifies the vasoconstrictor hyperreactivity to phenylephrine in mice with spontaneous atherosclerosis. Moreover, in healthy mice the oral *P gingivalis *infection also produces increased vasoconstrictor response to this α-adrenoceptor agonist. This finding supports the hypothesis that oral infection with *P gingivalis *is one of several risk factors of cardiovascular diseases.

## Competing interests

The authors declare that they have no competing interests.

## Authors' contributions

RBP conceived the study, carried out the animal experiments, analysis of data, statistics and drafted the manuscript. ECV and IS participated in the co-supervision of the study and in the critical revision of the manuscript. SSM participated in design and supervision and in the critical revision of the manuscript. All authors read and approved the final manuscript.

## References

[B1] Beck JD Offenbacher S Systemic effects of periodontitis: epidemiology of periodontal disease and cardiovascular disease J Periodontol 2005 76 2089 2100 10.1902/jop.2005.76.11-S.2089 16277581

[B2] Libby P Ridker PM Maseri A Inflammation and atherosclerosis Circulation 2002 105 1135 1143 10.1161/hc0902.104353 11877368

[B3] Gibson FC Hong C Chou HH Yumoto H Chen J Lien E Wong J Genco CA Innate immune recognition of invasive bacteria accelerates atherosclerosis in apolipoprotein E-deficient mice Circulation 2004 109 2801 2806 10.1161/01.CIR.0000129769.17895.F0 15123526

[B4] Lalla E Lamster Lb Hofmann MA Bucciarelli L Jerud AP Tucker S Lu Y Papapanou PN Schmidt AM Oral infection with a periodontal pathogen accelerates early atherosclerosis in apolipoprotein E-null mice Arterioscler Thromb Vasc Biol 2003 23 1405 1411 10.1161/01.ATV.0000082462.26258.FE 12816879

[B5] Li L Messas E Batista EL Jr Levine RA Amar S Porphyromonas gingivalis infection accelerates the progression of atherosclerosis in a heterozygous apolipoprotein E-deficient murine model Circulation 2002 105 861 867 10.1161/hc0702.104178 11854128

[B6] Loos BG Craandijk J Hoek FJ Wertheim-van Dillen PM van der Velden U Elevation of systemic markers related to cardiovascular diseases in the peripheral blood of periodontitis patients J Periodontol 2000 71 1528 1534 10.1902/jop.2000.71.10.1528 11063384

[B7] Slade GD Offenbacher S Beck JD Heiss G Pankow JS Acute-phase inflammatory response to periodontal disease in the US population J Dent Res 2000 79 49 57 10.1177/00220345000790010701 10690660

[B8] Noack B Genco RJ Trevisan M Grossi S Zambon JJ De Nardin E Periodontal infections contribute to elevated systemic C-reactive protein level J Periodontol 2001 72 1221 1227 10.1902/jop.2000.72.9.1221 11577954

[B9] Deshpande RG Khan MB Genco CA Invasion of aortic and heart endothelial cells by Porphyromonas gingivalis Infect Immun 1998 66 5337 5343 9784541 10.1128/iai.66.11.5337-5343.1998 PMC108667

[B10] Dorn BR Dunn WA Jr Progulske-Fox A Invasion of human coronary artery cells by periodontal pathogens Infect Immun 1999 67 5792 5798 10531230 10.1128/iai.67.11.5792-5798.1999 PMC96956

[B11] Amar S Gokce N Morgan S Loukideli M Van Dyke TE Vita JA Periodontal disease is associated with brachial artery endothelial dysfunction and systemic inflammation Arterioscler Thromb Vasc Biol 2003 23 1245 1249 10.1161/01.ATV.0000078603.90302.4A 12763762

[B12] Mercanoglu F Oflaz H Oz O Gökbuget AY Genchellac H Sezer M Nişanci Y Umman S Endothelial dysfunction in patients with chronic periodontitis and its improvement after initial periodontal therapy J Periodontol 2004 75 1694 1700 10.1902/jop.2004.75.12.1694 15732873

[B13] Seinost G Wimmer G Skerget M Thaller E Brodmann M Gasser R Bratschko RO Pilger E Periodontal treatment improves endothelial dysfunction in patients with severe periodontitis Am Heart J 2005 149 1050 1054 10.1016/j.ahj.2004.09.059 15976787

[B14] Elter JR Hinderliter AL Offenbacher S Beck JD Caughey M Brodala N Madianos PN The effects of periodontal therapy on vascular endothelial function: a pilot trial Am Heart J 2006 151 47 16368290 10.1016/j.ahj.2005.10.002

[B15] Higashi Y Goto C Jitsuiki D Umemura T Nishioka K Hidaka T Takemoto H Nakamura S Soga J Chayama K Yoshizumi M Taguchi A Periodontal infection is associated with endothelial dysfunction in healthy subjects and hypertensive patients Hypertension 2008 51 446 453 10.1161/HYPERTENSIONAHA.107.101535 18039979

[B16] Lopez JA Armstrong ML Piegors DJ Heistad DD Effect of early and advanced atherosclerosis on vascular responses to serotonin, thromboxane A_2 _and ADP Circulation 1989 79 698 705 2917393 10.1161/01.cir.79.3.698

[B17] Suwaidi JA Hamasaki S Higano ST Nishimura RA Holmes DR Jr Lerman A Long-term follow-up of patients with mild coronary artery disease and endothelial dysfunction Circulation 2000 101 948 954 10704159 10.1161/01.cir.101.9.948

[B18] Schächinger V Britten MB Zeiher AM Prognostic impact of coronary vasodilator dysfunction on adverse long-term outcome of coronary heart disease Circulation 2000 101 1899 1906 10779454 10.1161/01.cir.101.16.1899

[B19] Perticone F Ceravolo R Pujia A Ventura G Iacopino S Scozzafava A Ferraro A Chello M Mastroroberto P Verdecchia P Schillaci G Prognostic significance of endothelial dysfunction in hypertensive patients Circulation 2001 104 191 196 11447085 10.1161/01.cir.104.2.191

[B20] Heitzer T Schlinzig T Krohn K Meinertz T Münzel T Endothelial dysfunction, oxidative stress, and risk of cardiovascular events in patients with coronary artery disease Circulation 2001 104 2673 2678 10.1161/hc4601.099485 11723017

[B21] Gokce N Keaney JF Jr Hunter L Watkins MT Menzoian JO Vita JA Risk stratification for postoperative cardiovascular events via noninvasive assessment of endothelial function: a prospective study Circulation 2002 105 1567 1572 10.1161/01.CIR.0000012543.55874.47 11927524

[B22] Vita JA Keaney JF Jr Endothelial function: a barometer for cardiovascular risk? Circulation 2002 106 640 642 10.1161/01.CIR.0000028581.07992.56 12163419

[B23] Baker PJ Evans RT Roopenian DC Oral infection with Porphyromonas gingivalis and induced alveolar bone loss in immunocompetent and severe combined immunodeficient mice Arch Oral Biol 1994 39 1035 1040 10.1016/0003-9969(94)90055-8 7717884

[B24] Donovan J Brown P Blood collection Curr Protoc Immunol 2006 Chapter 1 Unit 1.7 10.1002/0471142735.im0107s73 18432965

[B25] Horne BD Anderson JL John JM Weaver A Bair TL Jensen KR Renlund DG Muhlestein JB Intermountain Heart Collaborative Study Group Which white blood cell subtypes predict increased cardiovascular risk? J Am Coll Cardiol 2005 45 1638 1643 10.1016/j.jacc.2005.02.054 15893180

[B26] Nogueira BV Peotta VA Meyrelles SS Vasquez EC Evaluation of aortic remodeling in apolipoprotein E-deficient mice and renovascular hypertensive mice Arch Med Res 2007 38 816 821 10.1016/j.arcmed.2007.06.005 17923260

[B27] Piedrahita JA Zhang SH Hagaman JR Oliver PM Maeda N Generation of mice carrying a mutant apolipoprotein E gene inactivated by gene targeting in embryonic stem cells Proc Natl Acad Sci USA 1992 89 4471 4475 10.1073/pnas.89.10.4471 1584779 PMC49104

[B28] Plump AS Smith JD Hayek T Aalto-Setälä K Walsh A Verstuyft JG Rubin EM Breslow JL Severe hypercholesterolemia and atherosclerosis in apolipoprotein E-deficient mice created by homologous recombination in ES cells Cell 1992 71 343 353 10.1016/0092-8674(92)90362-G 1423598

[B29] Zhang SH Reddick RL Piedrahita JA Maeda N Spontaneous hypercholesterolemia and arterial lesions in mice lacking apolipoprotein E Science 1992 258 468 471 10.1126/science.1411543 1411543

[B30] Zhang SH Reddick RL Burkey B Maeda N Diet-induced atherosclerosis in mice heterozygous and homozygous for apolipoprotein E gene disruption J Clin Invest 1994 94 937 945 10.1172/JCI117460 8083379 PMC295131

[B31] Jawień J Nastałek P Korbut R Mouse models of experimental atherosclerosis J Physiol Pharmacol 2004 55 503 517 15381823

[B32] Ross R Atherosclerosis - an inflammatory disease N Engl J Med 1999 340 115 126 10.1056/NEJM199901143400207 9887164

[B33] Miyamoto T Yumoto H Takahashi Y Davey M Gibson FC Genco CA Pathogen-accelerated atherosclerosis occurs early after exposure and can be prevented via immunization Infect Immun 2006 74 1376 1380 10.1128/IAI.74.2.1376-1380.2006 16428788 PMC1360301

[B34] Brodala N Merricks EP Bellinger DA Damrongsri D Offenbacher S Beck J Madianos P Sotres D Chang Yl Koch G Nichols TC Porphyromonas gingivalis bacteremia induces coronary and aortic atherosclerosis in normocholesterolemic and hypercholesterolemic pigs Arterioscler Thromb Vasc Biol 2005 25 1446 1451 10.1161/01.ATV.0000167525.69400.9c 15845905

[B35] Gimbrone MA Jr Endothelial dysfunction and atherosclerosis J Card Surg 1989 4 180 183 10.1111/j.1540-8191.1989.tb00275.x 2519996

[B36] Villeneuve N Fortuno A Sauvage M Fournier N Breugnot C Jacquemin C Petit C Gosgnach W Carpentier N Vanhoutte P Vilaine JP Persistence of the nitric oxide pathway in the aorta of hypercholesterolemic apolipoprotein-E-deficient mice J Vasc Res 2003 40 87 96 10.1159/000070705 12808344

[B37] Bonthu S Heistad DD Chappell DA Lamping KG Faraci FM Atherosclerosis, vascular remodeling, and impairment of endothelium-dependent relaxation in genetically altered hyperlipidemic mice Arterioscler Thromb Vasc Biol 1997 17 2333 2340 9409199 10.1161/01.atv.17.11.2333

[B38] Crauwels HM Van Hove CE Holvoet P Herman AG Bult H Plaque-associated endothelial dysfunction in apolipoprotein E-deficient mice on a regular diet. Effect of human apolipoprotein AI Cardiovasc Res 2003 59 189 199 10.1016/S0008-6363(03)00353-5 12829190

[B39] Ohashi M Runge MS Faraci FM Heistad DD MnSOD deficiency increases endothelial dysfunction in ApoE-deficient mice Arterioscler Thromb Vasc Biol 2006 26 2331 2336 10.1161/01.ATV.0000238347.77590.c9 16873728

[B40] Matsumoto T Miyamori K Kobayashi T Kamata K Apocynin normalizes hyperreactivity to phenylephrine in mesenteric arteries from cholesterol-fed mice by improving endothelium-derived hyperpolarizing factor response Free Radic Biol Med 2006 41 1289 1303 10.1016/j.freeradbiomed.2006.07.012 17015176

[B41] Gödecke A Ziegler M Ding Z Schrader J Endothelial dysfunction of coronary resistance vessels in apoE-/- mice involves NO but not prostacyclin-dependent mechanisms Cardiovasc Res 2002 53 253 262 10.1016/S0008-6363(01)00432-1 11744035

[B42] Xu X Gao X Potter BJ Cao JM Zhang C Anti-LOX-1 Rescues Endothelial Function in Coronary Arterioles in Atherosclerotic ApoE Knockout Mice Arterioscler Thromb Vasc Biol 2007 27 871 877 10.1161/01.ATV.0000259358.31234.37 17272755

[B43] Morikawa K Matoba T Kubota H Hatanaka M Fujiki T Takahashi S Takeshita A Shimokawa H Influence of diabetes mellitus, hypercholesterolemia, and their combination on EDHF-mediated responses in mice J Cardiovasc Pharmacol 2005 45 485 490 10.1097/01.fjc.0000159657.93922.cb 15821445

[B44] Arruda RM Peotta VA Meyrelles SS Vasquez EC Evaluation of vascular function in apolipoprotein E knockout mice with angiotensin-dependent renovascular hypertension Hypertension 2005 46 932 936 10.1161/01.HYP.0000182154.61862.52 16087779

[B45] Cola MS Gava AL Meyrelles SS Vasquez EC Endothelial dysfunction of resistance vessels in female apolipoprotein E-deficient mice Lipids Health Dis 2010 9 51 10.1186/1476-511X-9-51 20482882 PMC2886002

[B46] Fujioka D Nakamura S Yoshino H Shinohara H Shiba H Mizuno N Hasegawa N Shindoh N Uchida Y Ogawa T Kawaguchi H Kurihara H Expression of endothelins and their receptors in cells from human periodontal tissues J Periodontal Res 2003 38 269 275 10.1034/j.1600-0765.2003.00653.x 12753364

[B47] Ivey ME Osman N Little PJ Endothelin-1 signalling in vascular smooth muscle: pathways controlling cellular functions associated with atherosclerosis Atherosclerosis 2008 199 237 247 10.1016/j.atherosclerosis.2008.03.006 18436225

